# The Use of Disabled Insecticidal Proteins (DIPs) to Investigate the Interaction Between *Aedes aegypti*-Active Toxins from *Bacillus thuringiensis*

**DOI:** 10.3390/insects16111085

**Published:** 2025-10-23

**Authors:** Nelly Igwe, Neil Crickmore

**Affiliations:** School of Life Sciences, University of Sussex, Brighton BN1 9QG, UK

**Keywords:** Cry41Aa, Cry toxin receptor, entomopathogen

## Abstract

A variety of pesticidal proteins derived from the bacterium *Bacillus thuringiensis* exhibit activity against the yellow fever mosquito *Aedes aegypti* and are used to control this insect vector. Several of these proteins, including Cry1Ca and Cry2Aa, additionally have activity against lepidopteran insects. Furthermore, the specificity of Cry2Aa has recently been shown to depend on domain I of the Cry protein, whereas it is generally recognized that domain II is the primary specificity-determining domain. This work has made use of disabled forms of three Cry proteins (Cry2Aa, Cry1Ca and Cry11Aa) and one naturally non-active protein (Cry2Ab) in an in vivo competition assay to investigate whether Cry2Aa and the dual-active Cry1Ca share a common receptor with the other pesticidal proteins. It was found that despite their differing specificities and potential modes of action, all of the *Aedes*-active proteins tested made use of a common receptor, although evidence is presented that Cry2Aa can use multiple receptors. When additional toxins (Cry41Aa, Cry1Aa, Cry1Ac) with no activity against this mosquito were tested, they too were found to share the same receptor, suggesting that Cry toxins may have evolved to utilize a common set of receptors in insects but that additional factors determine species specificity.

## Simple Summary

The bacterium *Bacillus thuringiensis* produces protein toxins that are able to control the mosquito *Aedes aegypti*, which is a vector of several human diseases. Understanding the mechanism of action of these toxins is necessary for both prolonging the effectiveness of bioinsecticides based on this bacterium and to potentially create new products. We have used a relatively new approach involving mutated toxins to indicate that eight different toxins appear to bind to the same cell surface receptor in the target insect. This result not only enhances our knowledge of host–pathogen interactions in this particular system but also indicates a greater commonality in the evolution of these toxins than was previously appreciated.

## 1. Introduction

Pesticidal proteins produced by *Bacillus thuringiensis* and other bacteria have long been used as biological insecticides to target crop pests and vectors of human disease. These proteins are generally pore-forming toxins which act on the midgut tissues of the host following ingestion and can either kill the insect outright or allow the bacterium to invade and colonize [1]. Individual proteins show specificity to particular orders or species of insect and although several factors can influence this specificity, the primary determinant is based on the interaction of the toxin with a receptor on the surface of the midgut cells [2]. Loss or mutation of these receptors can result in the insect acquiring resistance to the toxin, and if multiple toxins share the same receptor, then cross-resistance is observed [3]. *Aedes aegypti* is a mosquito capable of transmitting a number of diseases such as dengue, yellow fever, chikungunya and Zika, and is able to spread further afield as a result of human movement and climate change [4]. Products based on strains of *B. thuringiensis* subsp. *israelensis* (Bti) have successfully been used for decades to control this pest; fortunately, the insect has not managed to evolve resistance to the bacterium [5]. It has been suggested that the lack of resistance to Bti is due to the fact that it produces multiple toxins that can act in synergy, and so multiple mutations would be required to induce a resistance phenotype [6]. Various researchers have attempted to identify the receptors utilized by the individual toxins of Bti. Alkaline Phosphatase (ALP) emerged as a strong candidate for the Cry11Aa toxin when a demonstration of binding between the two proteins was supplemented with the observation that knockdown of ALP1 by RNAi increased the tolerance of the insect larvae to Cry11Aa [7]. Subsequent work involving transgenic mosquitoes expressing a heat-inducible dsALP1RNA also observed a decrease in susceptibility to Cry11Aa upon induction [8]. However, a CRISPR-Cas9 generated knockout of ALP did not alter the insect’s susceptibility to either Cry11Aa, Cry4Aa or Cry4Ba [9]. That same study also found that knocking out the *A. aegypti* cadherin-like protein (CAD) did not alter susceptibility to the above three toxins. This seemed to contradict a previous finding that CAD could act as a receptor for Cry11Aa [10]. Regarding Cry4Ba, the ALP1 RNAi experiment described above [7] observed an increased tolerance to this toxin, whereas the inducible dsALP1RNA experiment [8] did not. Ectopic expression of ALP in Sf9 cells conferred susceptibility to Cry4Ba, and this strongly indicates its role as a functional receptor for this toxin [11]. Ectopic expression of two Aminopeptidase isoforms (AaeAPN2778 and AaeAPN2783) indicated that both could act as receptors for Cry4Ba, whereas the knocking out of one or both of two APN isoforms (APN1 and APN2) did not alter the susceptibility of larvae towards either Cry4Ba or Cry11Aa [12]. No clear picture has thus emerged concerning the functional receptors for the Bti toxins in *A. aegypti*. More recently, so-called Disabled Insecticidal Proteins (DIPs) have been used to investigate the receptor-binding preferences of Bt toxins [13]. These are mutated toxins that can no longer form pores but whose receptor-binding properties are largely unaltered. By performing in vivo competition assays, the effect of a DIP on the toxicity of a non-mutated toxin can indicate which toxins share common receptors. In this work we have used various DIPs to study the in vivo interactions between a number of different toxins with activity towards *A. aegypti*. In particular, we were interested in Cry2Aa. It has long been established that while Cry2Aa has activity against *A. aegypti,* the closely related Cry2Ab does not [14]. We identified that the differential specificity is associated with domain I of the protein [15,16], whereas specificity generally associates with domain II, and we wondered if this was reflected in Cry2Aa binding to a different type of receptor than the other toxins active against this mosquito.

## 2. Materials and Methods

### 2.1. Strains and Plasmids

We have previously described the expression systems used for Cry2Aa, Cry2Ab Cry1Ac, Cry1Ca, Cry11Aa, Cry4Aa and Cry41Aa [17,18]. For Cry1Aa and Cry4Ba, the gene encoding Cry2Aa in the respective plasmid was replaced with the open reading frames for these two other genes.

### 2.2. Toxin Preparation and Mosquito Bioassay

Recombinant Bt strains were grown in half-strength LB medium for 72 h at 30 °C, 180 rpm. Cells, spores and crystals were recovered by centrifugation, resuspended in water and sonicated to break open unlysed cells. Following further centrifugation (and a 2nd round of sonication if required), spores and crystals were resuspended in water. The purity and concentration of each toxin was assessed by SDS PAGE and densitometry, using BSA as a standard. To assess the likelihood of the introduced mutations affecting the structural instability of the toxins, all crystals were viewed under a phase-contrast microscope to check that the mutants maintained the same crystal shape as the wild-type. The solubilization of the crystals in alkali and the resistance of the solubilized toxin to digestion by 1 mg/mL trypsin was performed to confirm that the mutants had not undergone any significant structural changes. Mosquito assays were performed using third-instar larvae. Five larvae were placed in water in a 24-well cell culture plate. Toxin was added and the volume made up to 3 mL. 20 wells (ie 100 larvae) were used for each experiment. The larvae were incubated at 27 °C with a 16:8 light/day photoperiod for 24 h. For the in vivo competition assays, sufficient toxin was added to produce around 70% death and then various concentrations of competitor were added. For each experiment, the controls were wells with just the toxin, wells with no toxin and wells with the highest concentration of competitor. Experiments were abandoned if either of the latter two controls gave greater than 10% mortality. Assays were repeated at least three times to ensure consistent results. A competitor was considered to have completely inhibited the activity of a toxin if the relative mortality dropped below 20% at 100xfold excess. If the relative mortality dropped to 50–60%, but no lower, then inhibition was considered partial. To rule out non-specific effects, competition assays were also performed with excess amounts of BSA equivalent to the concentrations of the competitors used. BSA had no effect on the activity of the toxins.

## 3. Results

In order to undertake the in vivo competition assays, DIP variants of Cry2Aa and Cry1Ca were first created. The Cry2Aa DIP (G119C_N123A_L156C_R160A) was based on the equivalent Cry2Ab DIP [13] since these four amino acids are conserved between the two proteins. The Cry1Ca DIP (N98C_D143C) was also created based on a previously reported design [13]. Neither DIP was found to have any activity against *A. aegypti* larvae.

### 3.1. Cry2AaDIP and Cry2Ab Inhibit the Activity of Cry2Aa to Different Extents

**Figure 1:**
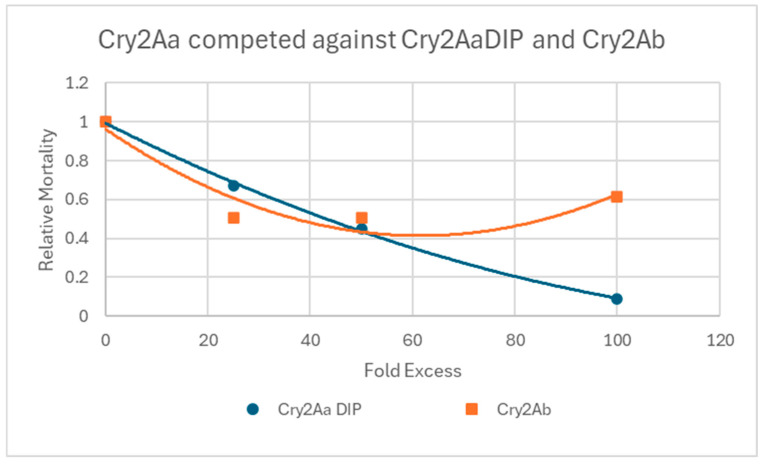
In vivo competition assay between Cry2Aa and Cry2Ab. Representative data normalized to the activity of Cry2Aa alone.

Figure 1 shows that when Cry2Aa DIP was fed to the larvae alongside Cry2Aa, there was a dose-dependent inhibition of activity. With a 100-fold excess of the DIP, an almost complete inhibition was observed. This observation is consistent with the model that DIPs are able to compete for binding with the native toxin but are not able to go on and cause toxicity. When present in large excesses they should displace virtually all binding of the native protein. In contrast, while excesses of Cry2Ab–which itself has no activity–caused some inhibition of Cry2Aa activity, they did not fully block activity. Such a result is consistent with the possibility that Cry2Aa can bind to multiple receptors, only one of which is blocked by Cry2Ab.

### 3.2. Interactions Between Other A. aegypti-Active Toxins

Although Cry1Ca is best known as a leipdopteran-active toxin, its activity against *A. aegypti* has been previously reported [19]. Because of this unusual activity for a Cry1 toxin, we were interested in how this would interact with more traditional mosquitocidal toxins. Cry11Aa, Cry4Aa and Cry4Ba are all toxins produced by the Bt *israelensis* strain used commercially to control mosquitoes and all three have known activity against *A. aegypti* [20]. As anticipated, the Cry1CaDIP completely inhibited the activity of Cry1Ca (Table 1). It also completely inhibited the activity of Cry4Ba and Cry11Aa. In contrast, but in common with the result seen above with Cry2Ab, Cry1CaDIP could only partially inhibit the activity of Cry2Aa. Both Cry2Aa DIP and Cry2Ab were able to completely inhibit the activity of Cry4Aa, Cry4Ba and Cry11Aa. These data suggest that the five active toxins all shared the same receptor(s), with Cry2Aa potentially having an additional one.

### 3.3. Cry11AaDIP and Cry41Aa Also Only Partially Inhibit Cry2Aa

A model in which Cry1Ca, Cry2Aa, Cry4Aa, Cry4Ba and Cry11Aa all share a common receptor would predict that a Cry11AaDIP would inhibit the activity of all these toxins—although for Cry2Aa, only partially. To test this hypothesis, the Cry11AaE100A variant was made based on a previous study of this mutant [21]. Table 1 shows that this DIP followed the same pattern as the other inactive proteins, in particular only partially inhibiting the activity of Cry2Aa. We had previously reported that the Cry41Aa Bt toxin with activity against some human cancer cells could block the activity of Cry1Ca in *A. aegypti* [18]. Since Cry1Ca appeared to use the same receptor as the other mosquitocidal toxins, we hypothesized that Cry41Aa would be able to also block the activity of these toxins, as indeed it could (Table 1).

### 3.4. Cry1Aa and Cry1Ac Could Also Inhibit the Activity of the Mosquitocidal Toxins

The observation that multiple Cry proteins—including two native toxins with no *A. aegypti* activity—could all inhibit the activity of the active toxins led us to wonder whether other toxins with no mosquitocidal activity would have the same effect. To test this, the lepidopteran-active Cry1Aa and Cry1Ac toxins were tested against Cry4Ba and were both found to completely inhibit its activity when present in excess.

## 4. Discussion

Although it is accepted that Bt toxins must bind to a cell surface receptor in order to efficiently go on and form a pore in the cell membrane, defining and identifying receptors is not straightforward. The first receptor to be identified was an aminopeptidase from *Manduca sexta* that was initially found as a binding partner of Cry1Ac [22]. A semi-purified preparation of this protein was also found to allow Cry1Ac-induced pore formation when incorporated into lipid vesicles [23]. In general, though binding per se is not a reliable indicator of receptor function since toxins have been shown to be able to bind to a wide range of different proteins, including intracellular ones such as actin that are extremely unlikely to be functional receptors [24]. Showing that RNAi of a putative receptor reduces the susceptibility to toxins [7] provides strong support, but it should be borne in mind that knockdown of non-receptor genes may also indirectly affect susceptibility [25]. More recently CRISPR-Cas9 knockouts have been widely used to characterize receptors [9] but perhaps the most powerful means of demonstrating receptor function is to show that ectopic expression in an otherwise non-susceptible cell makes that cell susceptible to the toxin [26]. Although the binding of toxins to other proteins is not always a reliable method for receptor identification, binding competition assays have long been used to predict whether different toxins might bind to the same receptor, and for determining the likelihood of cross-resistance [27]. To complement these in vitro binding assays, in vivo competition assays using Disabled Insecticidal Proteins (DIPs) have provided an additional means of investigating toxin–receptor interactions [13]. An underlying principle of these in vivo competition assays is that if a DIP form of a toxin inhibits the activity of a functional toxin, then it is assumed that they interact with the same receptor. Of course, other explanations are possible such as a direct interaction between the two toxin proteins. Based on the above principle, our data indicate that all of the toxins we tested against *Aedes aegypti* can bind to the same receptor, with the possibility that Cry2Aa can additionally bind to a different one. Whether this ability of Cry2Aa to bind to a different receptor is related to the role of its domain I in determining the differential activity of Cry2Aa and Cry2Ab to this mosquito [15,16,17] remains to be determined. Although all the toxins that we tested appear to bind to the same receptor, some of them (Cry1Aa, Cry1Ac and Cry41Aa) show no activity to *A. aegypti* despite being toxic to other hosts. There are numerous factors that can influence toxicity and specificity [2] but since competition for receptor binding has been observed, factors such as proteolytic instability or sequestration are unlikely to be the cause of non-toxicity. Although receptor binding is considered to be a pre-requisite for pore-formation in vivo, binding itself may not be sufficient for pore-formation or toxicity. It is quite feasible that a toxin can bind to a receptor in a way that prevents another toxin from binding to it, but that some aspect of this binding is not conducive to pore-formation. For example, if the binding position means that the pore-forming domain I is too far from or in the wrong orientation with respect to the membrane, then the ability to insert into the membrane may be hampered [28]. One of the reasons why Bti has proved to be such a successful biomosquitocide is that despite its extensive use, field resistance has not been consistently observed [29]. A strategy for delaying/preventing the development of resistance has been to use products containing multiple toxins that bind to different receptors. A target insect then has to acquire mutations in multiple receptors to show resistance [30]. It is somewhat surprising then that our data indicate that the three main Cry toxins of Bti (Cry11Aa, Cry4Aa and Cry4Ba) all utilize the same receptor. A separate hypothesis as to why mosquitoes have not developed resistance to Bti is the presence of the Cyt1Aa toxin which can interact and synergize with the Cyt1Aa toxin in a number of ways [31,32]. If indeed there is a single receptor for Cry11Aa, Cry4Aa and Cry4Ba in *A. aegypti*, it is not clear what that is. As discussed above, previous attempts to identify Cry toxin receptors in Bti have produced a confusing set of results.

## 5. Conclusions

The use of Disabled Insecticidal Proteins has indicated that multiple Cry toxins—both with and without activity towards *A. aegypti*—all share the same receptor in this mosquito. One of the toxins (Cry2Aa) may additionally utilize a second receptor. The observation that so many of the toxins can interact with the same receptor may reflect an evolutionary history in which Cry toxins bind to a common set of membrane proteins; however, subtle changes in the nature of this binding, or their ability to penetrate the membrane, has resulted in specificity towards particular insects.

## Figures and Tables

**Table 1:** In vivo competition results. The active toxins in the left-hand column (colored green) were competed against the proteins in the top row (colored pink). ND = not determined.

	Competitor	Cry2AaDIP	Cry1CaDIP	Cry2Ab	Cry11AaDIP	Cry41Aa
Toxin						
Cry2Aa		Complete	Partial	Partial	Partial	Partial
Cry1Ca		Complete	Complete	Complete	Complete	Complete
Cry4Aa		Complete	ND	Complete	Complete	Complete
Cry4Ba		Complete	Complete	Complete	Complete	Complete
Cry11Aa		Complete	Complete	Complete	Complete	Complete

## Data Availability

Dataset available on request from the authors.
